# Application of three-dimensional visualization technology in the anatomical variations of hilar bile ducts in Chinese population

**DOI:** 10.3389/fsurg.2022.934183

**Published:** 2022-08-02

**Authors:** Xiaofeng Li, Renpeng Duan, Yifeng He, Jiawei Qin, Ruijian Liu, Siqin Dai, Jiawei Zhou, Xiancheng Zeng, Juan Duan, Peng Gao, Xiaoqiao Yang, Cheng Li

**Affiliations:** ^1^ Guangdong Provincial Emergency Hospital, Guangdong Second Provincial General Hospital, Guangzhou, Guangdong, China; ^2^ The Second Affiliated Hospital of Guangzhou University of Chinese Medicine, Guangdong Provincial Hospital of Chinese Medicine, Guangzhou, Guangdong, China

**Keywords:** three-dimensional reconstruction, biliary tract anatomy, biliary tract disease, Hisense computer-assisted surgery system, China region

## Abstract

This study aimed to establish three-dimensional models of the biliary tract of Chinese people using the Hisense computer-aided surgery (CAS) system and to explore the branching patterns and variation types of the biliary system under the study of 3D reconstruction of the biliary tract. Three-dimensional models of the biliary tract were reconstructed in 50 patients using the Hisense CAS system. The branching patterns of intrahepatic bile ducts were observed. The biliary tract was classified according to the confluence of the right posterior sectoral duct (RPSD), right anterior sectoral duct (RASD) and left hepatic duct (LHD), and the presence or absence of accessory hepatic ducts. The 3D models of the bile ducts were successfully reconstructed in 50 Chinese patients. The branching patterns of the bile ducts were classified into seven types. The anatomy of the bile ducts was typical in 54% of cases (*n* = 27), showed triple confluence in 10% (*n* = 5), and crossover anomaly in 14% (*n* = 7), which means anomalous drainage of the RPSD into the LHD, anomalous drainage of the RPSD into the common hepatic duct (CHD) in 10% (*n* = 5), anomalous drainage of the RPSD into the cystic duct (CD) in 2% (*n* = 1), absence of left main hepatic duct in 1% (*n* = 1), presence of accessory duct in 8% (*n* = 4). Among them, there were three cases of accessory hepatic ducts coexisting with other variation types. By using the Hisense CAS system to establish 3D models of the biliary tract of the Chinese people, we established the branching model of the second-order bile ducts, which has important value for the classification of the biliary system and its variation types.

## Introduction

Variations in the anatomy of the biliary tract have been proven to be of clinical significance (1). Accurate knowledge of these anatomical variants is crucial for surgeons to avoid complications (2). Correct preoperative understanding of the bile duct variations may help to formulate a preoperative simulation and surgical planning, which can increase the success rate of surgery, reduce operation-related complications, and improve the prognosis of patients (3). Several reports have described the anatomy of the biliary tract, including studies on the human biliary system in different ways, such as casting research, autopsy specimens, endoscopic retrograde cholangiopancreatography (ERCP), Magnetic resonance cholangiopancreatography (MRCP), angiography, and detailed descriptions of the biliary anatomy with different classification systems (2, 4–7). However, there are few studies on the variations in biliary anatomy using 3D visualization technology. Three-dimensional visualization technology based on CT or MRI has been a research hotspot in recent years, and it has been widely and maturely applied in the field of hepatobiliary surgery. Nevertheless, it has rarely been used in the study of the biliary tract, mainly because the biliary system, in general, is not well developed on CT images, and thus, its complete structure cannot be objectively described by the reconstruction software. Harms et al. (8) studied donor liver for living donor liver transplantation using three-dimensional visualization technology. Zeng et al. (9) studied the application of three-dimensional visualization in hilar cholangiocarcinoma and reconstructed individualized branches of intrahepatic bile ducts. Li et al. (10) evaluated the application value of 3D reconstructed models and 3D printed models in the training of choledochoscopy techniques. None of them, however, systematically described the anatomic variation types of bile ducts. The advent of digital medicine and three-dimensional visualization software has improved surgeons’ ability to identify the biliary tract on CT images; this, combined with the imaging characteristics of obstructive biliary tract diseases, has created conditions for the three-dimensional reconstruction of the biliary tract.

The Hisense computer-aided surgery (CAS) system, a three-dimensional reconstruction software based on CT images, can realize in-depth mining of CT image information and reproduce the anatomy of abdominal organs and vessels accurately. A solid understanding of individualized anatomic differences of the biliary system informs both the planning of the procedure and intraoperative decision-making. In the present article, we perform 3D modeling based on CT imaging data from patients with obstructive disease of the bile ducts using the Hisense CAS system to show the normal and abnormal anatomy of biliary trees of Chinese people based on the three-dimensional visualization technology; meanwhile, we analyze the branching pattern and variation types of Chinese biliary tract according to the three-dimensional reconstructed models.

## Materials and methods

### Research objects

From September 2019 to December 2020, 80 patients with obstructive disease of the bile ducts admitted to the Second People's Hospital of Guangdong Province were evaluated for study eligibility. These patients underwent enhanced CT scans of the upper abdomen. In that, 30 of them were excluded due to poor image quality or obscuration of biliary anatomy. Among them, 13 patients with poor cooperation resulted in more CT image artifacts, which affected bile duct reconstruction, and 17 patients had insufficient or uneven bile duct dilation, which affected bile duct alignment recognition. Hence, our final study group included 50 cases (30 males and 20 females; age range 29–85 years). With the approval of the ethics committee, all patients signed an informed consent form.

### Inclusion criteria

The inclusion criteria included: (1) patients with clear CT images and well-dilated branches of the biliary tract below grade 2 (diameter greater than 0.5 mm); (2) patients from China. The exclusion criteria included: (1) patients with blurry CT images or CT artifacts, and patients whose intrahepatic bile ducts cannot be identified; (2) patients with a history of bile duct surgery, bile duct injury, hepatectomy, and liver transplantation.

### CT data acquisition

CT scan images were obtained using a 256-slice spiral CT (Brilliance 256, Philips Healthcare, Best, The Netherlands), including plain scan and enhanced phase data. A non-ionic contrast agent (iopamidol, 100 ml: 37 g(I)) was used for enhanced scanning (arterial phase, portal phase, and venous phase). The software used for data processing and 3D reconstruction includes the RadiAnt DICOM Viewer and the Hisense CAS system. Patients orally took 500–1000 ml of clear liquid 20–30 min before the examination, and then another 500 ml before the start of the scan to fill the gastrointestinal tract (as a negative contrast agent). Patients were trained to hold their breath in full inspiration to maximize the control of artifacts due to respiratory motion, and took a supine scan from the top of the diaphragm to the lower edge of the liver. The acquisition parameters were set as follows: tube current, 300 mA; tube voltage, 120 kV; slice thickness, 1.0 mm; pitch, 0.993; and scan speed, 0.5 s/rotation. The plain scan is a high-resolution volume scan in the sub-millimeter state. A double-barrel high-pressure syringe was used, and the contrast agent bolus (dose 1.5 ml/kg) was injected into the cubital vein at a speed of 5 ml/s. The scan delay time after the arterial phase injection was 20–25 s, and the scan delay time after the intravenous phase injection was 50–55 s. After scanning, the image data were transferred to the postprocessing workstation and burned to a disc for storage.

### 3D reconstruction

The original 1 mm thin-slice CT scan data from the burned CD were extracted, including data in the plain scan phase, arterial phase, venous phase, and delayed phase. Collected data were converted into DICOM format by using a Radiant Dicom Viewer and then imported into the Hisense CAS system. In the mode of “3D reconstruct”, the appropriate phase of CT scan was selected, and the window width and window level were adjusted to perform 3D reconstruction of liver parenchyma, hepatic vascular system, bile duct system, and calculi, respectively. The reconstructed data in different phases were marked with different colors, and then a complete hepatobiliary system was constructed. The three-dimensional model of the liver and bile ducts was hidden, rotated, zoomed in, and transparent with the CAS system, and the three-dimensional model of the bile ducts was separately displayed to study the anatomy and variation of the bile ducts. Meanwhile, the length of the right hepatic duct (RHD) was also measured in three dimensions. The three-dimensional reconstruction software (the CAS system) used in this study was purchased by our research team at a cost of approximately 700,000 RMB. The software is mainly used for clinical research and preoperative evaluation. It can be used repeatedly, and the cost of each reconstruction in the later stage is about 3,000 RMB. The entire reconstruction process was performed by the personnel who have obtained the training certificate for system reconstruction. For cases of different complexity, it takes about 30–60 min to complete the entire reconstruction process.

### Data analysis

The 3D models were reviewed by a senior consultant radiologist and a senior hepatobiliary surgeon. Anatomic variations were classified according to the Huang classification (2), based on the confluence of the RPSD, RASD, and LHD. SPSS statistical analysis software was used to calculate the type of biliary anatomy and the percentage analysis of the model in southern China; moreover, an appropriate chi-square test was used to test whether gender and variation are related, and a *P* less than 0.05 indicates statistical significance.

### Ethical statement

The study protocol was approved by the ethics committee of the Guangdong Second Provincial General Hospital and written informed consents were obtained from all patients to undergo the study. The study application form and ethical review form are shown in the additional materials.

## Results

**Figure 1:**
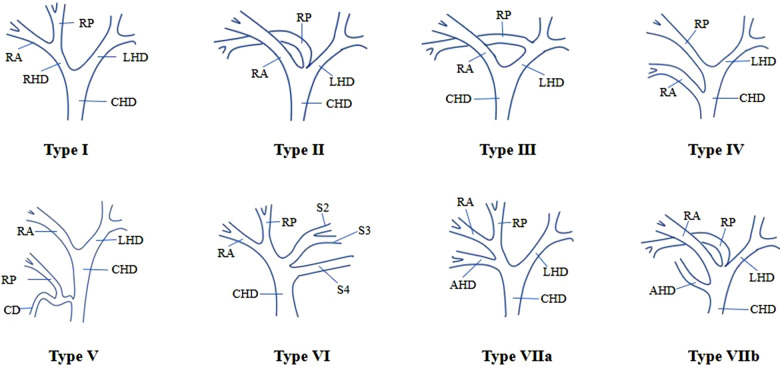
Intrahepatic bile duct anatomy based on three-dimensional biliary tract reconstruction. Type I shows a typical anatomical structure of the biliary tract. Type II is triple confluence, i.e., RASD, RPSD, and LHD confluence into the CHD. Type III shows abnormal RPSD drainage to LHD, abnormal RPSD drainage to the CHD, and abnormal RPSD drainage to the CD. In type VI, there is no true LHD, and the bile duct draining segment 4 and the bile duct draining segments 2 and 3 are inserted into the CHD, respectively. Type VII has AHD, and it can be divided into type VIIa and type VIIb, according to the opening position of AHD. In type VIIb, there are also type II variants besides the opening of AHD in the CHD. RPSD = right posterior segmental duct, RASD = right anterior segmental duct, RHD = right hepatic duct, LHD = left hepatic duct.

**Figure 2:**
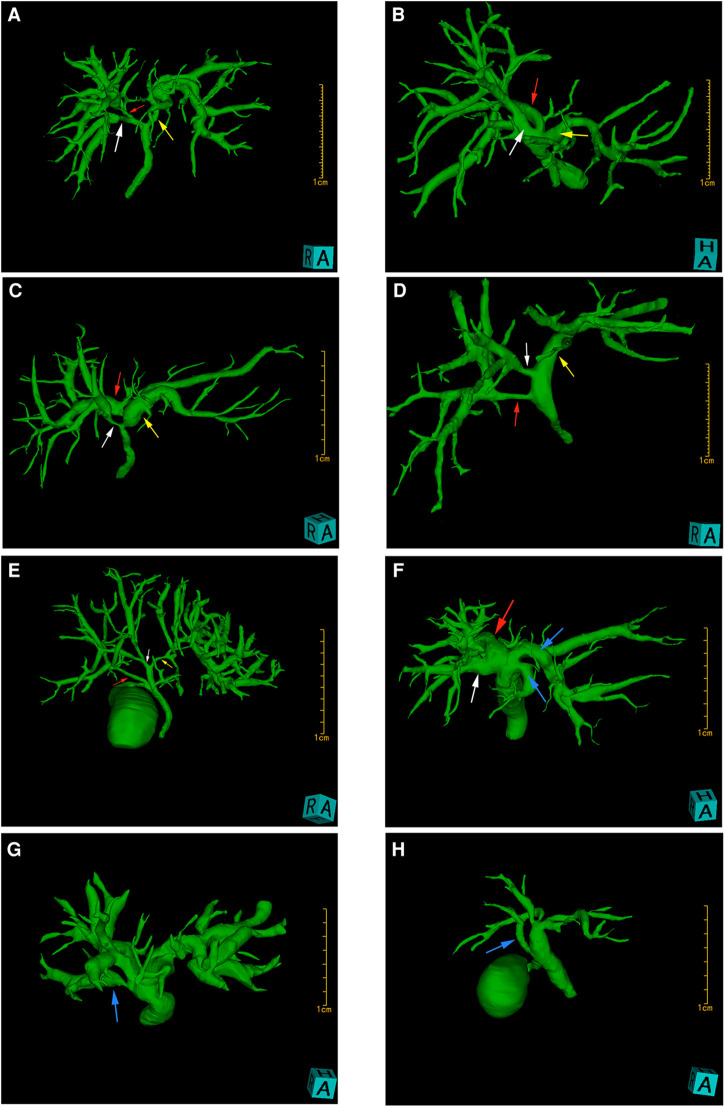
3D biliary tract model reconstructed by the Hisense CAS system. The white arrowheads indicate RASD, the red arrowheads indicate RPSD, the yellow arrowheads indicate LHD, and the blue arrowheads indicate variant bile ducts. (**A**) shows typical intrahepatic bile duct anatomy (type I), i.e., RASD (white arrowhead) forms RHD with RPSD (red arrowhead), which in turn forms CHD with LHD. The bile duct shown in (**B**) is classified as type II, indicating triple confluence, i.e., RASD, RPSD, and LHD drain into the CHD. (**C**) is a type III variant showing abnormal RPSD drainage to LHD. (**D**) shows an abnormal RPSD drainage to CHD, and (**E**) presents a rare variant showing RPSD drainage to the cystic duct, and is classified as type V. In (**F**), the bile duct draining segment 4 (blue arrowhead) and the bile duct draining segments 2 and 3 (blue arrowhead) are inserted into CHD, respectively, and there is no left main hepatic duct in this variant. Accessory hepatic ducts (blue arrowhead) are present in both (**G**) and (**H**), which are classified as type VIIa and type VIIb, respectively, according to the opening position of the accessory hepatic ducts. In type VIIa, AHD is inserted into RHD, not accompanied by other types of variations, while in type VIIb, AHD can be observed opening into the hepatic duct, accompanied by a variation of type II.

Three-dimensional reconstruction of the liver parenchyma, intrahepatic and extrahepatic bile ducts, and intrahepatic vessels was completed in 50 Chinese patients using the Hisense CAS system. The reconstructed hepatobiliary system was complete, which clearly showed the 3D structure of the intrahepatic ducts. Spatial configuration and abnormalities of these ducts could be observed by zooming in, zooming out, and all-around rotation. By hiding and transparency adjustment of the liver and intrahepatic vascular system, a three-dimensional model of the bile duct of Chinese people was obtained. The model was rotated and observed. The classification was conducted according to Huang's classification (2), based on the confluence of the RPSD, RASD, and LHD. The branching patterns were classified into seven types (Figure 1). A total of 27 patients (54%) had typical biliary tract anatomy (type I) (Figure 2A). Anatomic variations were found in 23 patients (46%), including five patients (10%) with type II (Figure 2B), seven patients (14%) with type III (Figure 2C), five patients (10%) with type IV (Figure 2D), one male patient (2%) with type V (Figure 2E), and one patient (2%) with type VI (Figure 2F); in addition, four cases (8%) had accessory hepatic ducts, among which three cases were accompanied by other variations (Figure 2H and I). The variation types and frequencies of the 50 cases in the three-dimensional model of the bile duct are shown in Table 1.

**Table 1:** Biliary tract anatomy and variation frequency based on three-dimensional visualization.

Type	Description of variant	Males	Females	Total
I	RASD forms RHD with RPSD, which in turn forms CHD with LHD	14 (46%)	13 (65%)	27 (54%)
II	Triple confluence, RASD, RPSD, and LHD drain into CHD	3 (10%)	2 (10%)	5 (10%)
III	RPSD drains into LHD	4 (13.3%)	3 (15%)	7 (14%)
VI	RPSD drains into CHD	4 (13.3%)	1 (5%)	5 (10%)
V	RPSD drains into the cystic duct	1 (2%)	0 (0%)	1 (2%)
VI	Bile ducts draining segment 4 and bile duct draining segments 2 and 3 were inserted into CHD, respectively	1 (2%)	0 (0%)	1 (2%)
VII	AHD, with or without other variations	3 (10%)	1 (5%)	4 (8%)
Total		30 (60%)	20 (40%)	50 (100%)

RPSD, right posterior segmental duct; RASD, right anterior segmental duct; RHD, right hepatic duct; LHD, left hepatic duct; CHD, common hepatic duct; AHD, accessory hepatic duct.

## Discussions

There are four sets of ductal systems in the liver, each with complex structures, among which the biliary tract has the highest rate of anatomical variations. Preoperative awareness of the pattern of these variations may help to prevent and manage biliary injuries during surgical procedures. Several imaging modalities for the evaluation of the biliary tract *in vivo* can be used. ERCP and percutaneous transhepatobiliary tract (PTC), as the gold standard for this purpose, can accurately show the course of the biliary tract; however, they are rarely used preoperatively because they are invasive methods and prone to complications such as acute pancreatitis and cholangitis (11). MRCP, as a standard and non-invasive examination method for evaluating the biliary system, can present a clear anatomical structure of the biliary system without the use of contrast agents and has a high sensitivity in the identification of variations (12), and 3D-MRCP, in particular, can provide relatively high-quality three-dimensional images, which has a high application value in the evaluation of biliary tract structure (13); nevertheless, disadvantages of MRCP include decreased spatial resolution, making MRCP less sensitive to abnormalities of peripheral intrahepatic duct (6), and thus, its efficacy in preoperative evaluation of biliary tract diseases and its guiding significance for biliary tract surgery is limited (14). In addition, the potential problems with MRCP also include image artifacts and difficulty in patient compliance, so it is generally not used as a preoperative examination for routine surgery (13). Some studies (15) even reported that MRCP was not effective in describing the branching pattern of the second-order bile duct and was insufficient to detect the variation of the bile duct preoperatively. As a non-invasive method, CT is a useful modality for the preoperative assessment of the biliary system, and its data acquisition is relatively simple and convenient. In this study, based on the CT image data, the Hisense CAS system was used to establish three-dimensional models of the biliary tract and identify the type of variation, which could help perform preoperative simulation programs, develop the best operation plans, and improve the accuracy and safety of actual surgery (16).

3D visualization technology is based on the reconstruction of CT image data. The reconstructed 3D model can provide more intuitive and comprehensive information on liver parenchyma and intrahepatic duct system and can be adjusted with different transparency and rotation angle so that surgeons can observe the anatomical structure of the bile duct from multiple directions and angles. Anatomic variation and lesion location of the biliary tract can be accurately identified (17). The three-dimensional visualization software can place the liver parenchyma, vascular system, and bile duct system in the same space, and display the spatial position relationship between the liver, intrahepatic vessels, and bile duct and lesions stereoscopically (18), which has an important guiding significance for hepatobiliary surgery. In addition, with the development of virtual reality technology and 3D printing technology, the reconstructed 3D model is applied to the preoperative simulation operation, making the actual operation safer and less traumatic (19, 20). Obstructed biliary tract disease leads to the dilation of intrahepatic and extrahepatic bile ducts, and the bile duct system is fully shown on CT images, which is significantly different from the CT values of other liver tissues. Based on this imaging feature, the present study can reconstruct a complete three-dimensional model of the bile duct system by using the Hisense CAS system. Its hidden and transparent functions can be used to display the biliary tract system in stereo. By rotating and zooming in, the anatomical structure of the biliary tract system can be observed from all angles and the variation of the biliary tract can be identified.

Anatomic variations of the biliary tract have been described by several classification systems (21). The frequency of biliary tract variations is influenced by region and ethnicity. Typical biliary anatomy is more common in Asians than in Europeans and Americans, probably because of the difference in embryo development, and a certain correlation with gender, as variations of the biliary tract are more common in females (22). Such findings were also reported in a study of MRCP in 1,011 inhabitants of the Aegean region of Turkey (23). However, in a study report of 150 Saudi Arabian population, no gender difference was detected (24). Similarly, in our study, Fisher's accurate test showed no significant correlation between the anatomic variations of the biliary tract and gender (*P* > 0.05). Although several earlier studies on biliary anatomy have varied in terms of research methods and ethnicity of the population studied, the results generally showed that the typical biliary anatomy (type I) was the most common biliary structure, occurring in approximately 55% to 79% of cases. Among the variant types, the right posterior hepatic duct (RPHD) drainage to the LHD (type III) was the most common, noted in about 9.3% to 28.7%. The so-called “trifurcation pattern” (type II) is the second most common, noted in about 9%–19%, and the insertion of the RPHD into the CHD or CD is the least common (4–6, 21, 23, 24). The Hisense CAS system analysis results showed that type I accounted for 54%, which was the typical biliary anatomical structure, types II and III were the most common variant types, accounting for 10% and 14% respectively, and types IV and V were the least common variant types, accounting for 10% and 2% respectively; these findings were similar to earlier literature reports (4–6, 21, 23, 24). In addition, we were surprised to note that some of the specific biliary tract types could not be explained according to the Huang classification. The bile duct variations shown in Figure 2F are defined as “type VI”, with a variation rate of 2%. This type of variation is described as the bile duct draining into the hepatic duct at segment 4 and the bile duct draining into segments 2 and 3, respectively, which is very similar to type 6 in Choi's classification system (4), i.e., the bile duct draining into the hepatic duct at segments 2 and 3, respectively. Accessory hepatic duct (AHD) is usually defined as the bile duct that merges with the extrahepatic bile duct and drains a certain segment of the liver or liver lobe. The report of its incidence varies widely in different literature, ranging from 1.9% to 31.4% (25). In our study, four cases (8%) had accessory hepatic ducts, of which one case was alone, two cases were accompanied by type II variation, and one case was accompanied by type VI variation. According to the classification proposed initially by Huang, the length of the RHD of type I was required to be greater than 1 cm. We used the Hisense CAS system to conduct three-dimensional measurements of the three-dimensional model of the biliary duct, and the results showed that in all the subtype biliary duct reconstruction models, five cases had the length of the RHD less than 1 cm. According to the classification, the type I prevalence decreases to 48%, which is still the most common type.

Preoperative assessment of the biliary tract anatomy plays a pivotal role in guiding surgery. In living donor right hepatectomy, if the type I bile duct has a longer RHD in the donor's liver, the difficulty of end-to-end bile duct anastomosis or biliary enterostomy can be reduced. When performing left hepatic resection on patients with type III bile duct, the greater the distance between RPHD insertion and the RHD and LHD junction, the more likely it is to damage or ligate the right posterior bile duct when the variation is unknown, resulting in hepatic cholestasis, cirrhosis, or bile leakage of the entire liver segment drained by the right posterior bile duct. In percutaneous transhepatic biliary drainage, if the LHD obstruction occurs in patients with type III bile duct, cholestasis in the right posterior lobe of the liver may occur. In addition to drainage of the left half of the liver, drainage of the right posterior lobe of the liver is also required. In percutaneous transhepatic choledochoscopic removal of stones, the selection of the lithotomy sinus tract directly affects the quality and efficiency of the operation, which should be determined according to the shape of the biliary tract. The most reasonable lithotomy fistula should be able to explore the stones without tearing the liver and biliary tract due to the excessive angle between the long axis of the choledochoscope and the lithotomy sinus tract. In laparoscopic cholecystectomy, anatomical abnormality of the bile duct is considered one of the risk factors for bile duct injury, with an incidence of 0.5%–1.7% (26). During laparoscopic cholecystectomy, attention should be paid to the existence of accessory hepatic ducts and types IV and V variations, because the Calot's triangle is covered by a thick layer of adipose tissue, and the lower insertion of the right posterior bile ducts or accessory hepatic ducts is prone to damage during separation. With preoperative knowledge regarding the existence of these variations, the vigilance of surgeons in this surgical area can be increased, and the ducts at Calot's triangle can be carefully isolated during operation to reduce the risk of iatrogenic bile duct injuries.

## Prospects

The limitation of our study was that the selected subjects are all obstructive biliary tract diseases, and hence the results are biased, though the results are similar to other studies. In addition, sufficient reconstruction data should be included to more accurately describe the frequency of biliary anatomical variations in populations in southern China. We look forward to discovering new types of biliary variations based on three-dimensional visualization of the biliary tract and then combining them with the practice of hepatobiliary surgery, so as to propose a more suitable classification system.

## Conclusions

Through the Hisense CAS system, we re-verified that type I remains the most common type in the Chinese population, while types II and III are the most common variants; in addition, we found that some specific biliary tract types defined as “type VI” could not be explained according to the Huang classification. By using 3D visualization technology to establish 3D models of the biliary tract of the Chinese people, we established the branching model of the second-order bile ducts, which has significant value for the classification of the biliary system and its variation types and for the reduction of postoperative complications.

## Contribution to the field

There are four sets of ductal systems in the liver, each with complex structures, among which the biliary tract has the most anatomical variations. Preoperative awareness of the pattern of these variations may help to prevent and manage biliary injuries during surgical procedures. Several imaging modalities for the evaluation of the biliary tract *in vivo* can be used. ERCP and PTC, as the gold standards for this purpose, can accurately show the course of the biliary tract; however, they are rarely used preoperatively because they are invasive methods and prone to complications such as acute pancreatitis and cholangitis. As a non-invasive method, CT is applied as a useful modality for the preoperative assessment of the biliary system, and data acquisition is relatively simple and convenient. Moreover, preoperative assessment of the biliary tract anatomy plays a pivotal role in guiding surgery. In this study, based on the CT image data, the Hisense CAS system was used to establish three-dimensional models of the biliary tract and identify the type of variation, which could help perform preoperative simulation program, develop the best operation plans, and improve the accuracy and safety of actual surgery.

## Data Availability

The original contributions presented in the study are included in the article/Supplementary Material, further inquiries can be directed to the corresponding author/s.
